# Academy of Stomatology—Resolutions upon the Death of Dr. Bonwill

**Published:** 1900-01

**Authors:** 


### ACADEMY OF STOMATOLOGY—RESOLUTIONS UPON THE DEATH OF DR. BONWILL.

The committee on resolutions upon the death of Dr. Bonwill beg leave to submit the following:

Whereas, W. G. A. Bonwill, D.D.S., a member of the Academy of Stomatology, has been removed by death, it becomes our mournful pleasure to make record of his worth; therefore be it

*Resolved*, As the sense of this society, that in the death of Dr. Bonwill the Academy has lost a distinguished member and the dental profession one of its best-known followers.

As a man Dr. Bonwill was genial and affable, though often misunderstood. As a dentist he was skilful and conscientious. As an inventor he had no superior in the dental profession. As an enthusiastic worker in the field of dental advancement he had few equals.

Entering upon the study of dentistry at an early age and under pecuniary disadvantages, he worked his way to success and eminence by burning zeal and untiring industry. His temperament was such that he could not be idle, and while others slept he was awake and working out problems which have made his name famous throughout the dental world.

As fellow-comrades, marching to the eternal world, we shall miss Dr. Bonwill from our ranks. Let us therefore loiter for a moment on the busy highway of life to hang one garland on his tombstone.

*Resolved*, That a copy of these resolutions be engrossed upon the records of the Academy and additional copies sent to his family and the dental journals.

Edwin T. Darby, *Chairman*,
James Truman,
I. N. Broomell,
Harry B. Hickman, *Secretary*,
*Committee*.

